# Factor H-related protein 1 in systemic lupus erythematosus

**DOI:** 10.3389/fimmu.2024.1447991

**Published:** 2024-07-29

**Authors:** Jessica S. Kleer, Juliane Klehr, Denise Dubler, Laura Infanti, Carlo Chizzolini, Uyen Huynh-Do, Camillo Ribi, Marten Trendelenburg

**Affiliations:** ^1^ Laboratory of Clinical Immunology, Department of Biomedicine, University of Basel, Basel, Switzerland; ^2^ Division of Internal Medicine, University Hospital, Basel, Switzerland; ^3^ Regional Blood Transfusion Service, Swiss Red Cross, Basel, Switzerland; ^4^ Department of Pathology and Immunology, University Hospital, Geneva, Switzerland; ^5^ Department of Nephrology and Hypertension, University Hospital, Bern, Switzerland; ^6^ Division of Immunology and Allergy, Department of Internal Medicine, University Hospital, Lausanne, Switzerland

**Keywords:** complement system, alternative pathway, Factor H (FH), Factor H-related proteins (FHRs), systemic lupus erythematosus (SLE), FHR1 deficiency

## Abstract

**Background:**

Factor H (FH) is a major soluble inhibitor of the complement system and part of a family comprising five related proteins (FHRs 1–5). Deficiency of FHR1 was described to be linked to an elevated risk of systemic lupus erythematosus (SLE). As FHR1 can partially antagonize the functionality of FH, an altered FHR1/FH ratio could not only enhance SLE vulnerability but also affect the disease expression. This study focuses on the analysis of FH and FHR1 at a protein level, and the occurrence of anti-FH autoantibodies (anti-FH) in a large cohort of SLE patients to explore their association with disease activity and/or expression.

**Methods:**

We assessed FH and FHR1 levels in plasma from 378 SLE patients compared to 84 healthy controls (normal human plasma, NHP), and sera from another cohort of 84 healthy individuals (normal human serum, NHS), using RayBio^®^ CFH and CFHR1 ELISA kits. Patients were recruited by the Swiss SLE Cohort Study (SSCS). Unmeasurable FHR1 levels were all confirmed by Western blot, and in a subgroup of patients by PCR. Anti-FH were measured in SLE patients with non-detectable FHR1 levels and matched control patients using Abnova’s CFH IgG ELISA kit.

**Results:**

Overall, FH and FHR1 levels were significantly higher in healthy controls, but there was no significant difference in FHR1/FH ratios between SLE patients and NHPs. However, SLE patients showed a significantly higher prevalence of undetectable FHR1 compared to all healthy controls (35/378 SLE patients versus 6/168 healthy controls; p= 0.0214, OR=2.751, 95% CI = 1.115 – 8.164), with a consistent trend across all ethnic subgroups. Levels of FH and FHR1, FHR1/FH ratios and absence of FHR1 were not consistently associated with disease activity and/or specific disease manifestations, but absence of FHR1 (primarily equivalent to CFHR1 deficiency) was linked to the presence of anti-FH in SLE patients (p=0.039).

**Conclusions:**

Deficiency of FHR1 is associated with a markedly elevated risk of developing SLE. A small proportion of FHR1-deficient SLE patients was found to have autoantibodies against FH but did not show clinical signs of microangiopathy.

## 1 Introduction

Systemic Lupus Erythematosus (SLE) is a chronic, multisystem autoimmune disorder characterized by a striking heterogeneity of clinical presentations, accompanied by hematological and serological abnormalities, such as reduced complement levels and elevated autoantibodies to various self-antigens. The etiology of SLE involves complex interactions between genetic and environmental factors; however, the exact underlying pathophysiological processes remain elusive (1).

The complement system (CS) is a central part of the innate immunity and serves as a first line of defense against foreign and altered host cells (2). It is strongly implicated in SLE, where it is believed to contribute to inflammation and tissue damage (3). The CS can be activated via three pathways; the classical (CP), the lectin (LP) and the alternative pathway (AP). Unlike the CP or LP, the AP does not require any specific surface recognition molecule for activation. Instead, the AP initiates spontaneously by the hydrolysis of C3 to C3(H2O) in the fluid phase, leading to the generation of C3b molecules that bind to nearby surfaces (4). These C3b molecules are not only the base for the formation of the AP C3 convertase (C3bBb) that cleaves additional C3 molecules into C3b and thereby amplifies the complement response, but also act as opsonins that tag cells for phagocytosis (5). This ‘always on’ positive feedback loop of C3b amplification is designed to rapidly opsonize invading microbes while being tightly regulated on autologous surfaces by protective, membrane-bound, and soluble regulators to prevent accidental host tissue damage (6–10).

Factor H (FH) is the main fluid phase regulator of the AP, recognizing host surfaces to inhibit accidental deposition of C3b (10). It is part of a protein family that includes FH-like protein (FHL-1) and five FH-related proteins (FHRs; FHR-1–5) (11). The FH gene family is located within the regulators of complement activation (RCA) gene cluster on chromosome 1q31 and consists of six genes arranged in tandem: CFH-CFHR3-CFHR4- CFHR2- CFHR5 (12). Phylogenetically, the CFHRs have evolved from CFH though genomic duplication events, making the CFH–CFHRs loci prone to genetic structural rearrangement (13). The secreted protein products of these genes are related in structure and consist of repetitive units named short consensus repeats (SCRs) of ~ 60 amino acids. FH’s complement regulatory function is mediated by the first four N-terminal SCRs, whereas SCRs 6–8, and SCR 19–20 are involved in surface recognition (14–16). The main conservation between FH and the FHRs lies within the surface recognition domains, which enables these proteins to bind similar ligands on surfaces, such as heparin or C3b (17). In contrast, the N-terminal SCRs 1 and 2 of FHR1, FHR2 and FHR5 contain a shared dimerization motif, hence these proteins circulate as either homo- or heterodimers (18). Unlike FH, the FHRs lack equivalent SCRs that mediate complement regulatory activities. The *in vivo* function of the FHRs is poorly characterized. However, recent data suggest that FHRs have indirect complement activation properties by competing with FH for ligand and surface binding, thus functioning as potential regulators of complement activation (19).

The significance of FHRs in various diseases has been underscored through genetic studies (20–23) yet the protein levels of the FHRs in these conditions remain poorly characterized (24). Zhao et al. demonstrated an association of the common deletion of CFHR3 and CFHR1 with an elevated risk for SLE (23), suggesting that lower levels of FHR1 and FHR3 increase the risk of SLE. FHR1, being the most abundant and best-studied FHR, is proposed as the most significant *in vivo* antagonist of FH due to its C-terminal SCRs closely resembling SCR 18–20 of FH (25). Dimerization of FHR1 increases avidity of the molecules (18) and could enhance their capacity for competing with FH. However, FHR1 does not recognize sialic acids—a key property of the FH C-terminus—due to two amino acid differences between the last two SCRs of FH and those of FHR1 (26).

This study aimed to evaluate FHR1 as a potential biomarker in SLE by determining the levels of FH and FHR1 and assessing their relationship with clinical manifestations of the disease. By characterizing the levels of FHR1 in SLE, we aim at digging into complement pathogenic mechanisms and contribute at clarifying the still ambiguous function of the FHR proteins.

## 2 Materials and methods

### 2.1 Patients and controls

To determine the association of FH and FHR1 levels with SLE disease manifestations, plasma samples and related clinical data from 378 SLE patients were provided by the Swiss SLE Cohort Study (SSCS), as described previously (27). As reference, we used plasma of 84 healthy blood donors (normal human plasma, NHP) and serum of additional 84 healthy blood donors (normal human serum, NHS) which were obtained from the blood donation center in Basel.

### 2.2 FH and FHR1 ELISA

FH and FHR1 levels were measured using the CFH and CFHR1 ELISA kits from RayBiotech (Peachtree Corners, GA, USA). According to the manufacturer, the CFH ELISA antibody pair specifically detects human FH. Other molecules, including FHL1, are not detected by the antibody pair. The lower limit of detection (LOD) was calculated using the formula $LOD=\frac{3.3\;\times \sigma }{S}$ , where σ is the standard deviation of the zero-standard response, and S is the slope of the calibration curve derived from linear proportion of the curve up to 4ng/ml. For FHR1, an LOD of 0.6 ng/ml was calculated. For data analysis, any FHR1 concentration measured below this value was classified as ‘Below LOD’. In the CFHR1 ELISA, the calibration curve was established up to 100 ng/ml. With regard to the upper limit of detection ODs were interpolated to estimate higher concentrations using Prism software. Using a dilution factor of 3000, the extrapolated values reached up to 376 µg/ml.

### 2.3 Anti-FH ELISA

FHR1-deficient SLE patients and matched non-deficient SLE controls were tested for anti-FH IgG using a commercially available ELISA kit (CFH IgG ELISA kit; Abnova, Heidelberg, Germany). The anti-FH titer was expressed in arbitrary units per mL (AU/mL). The cutoff value for anti-FH positivity was determined using the mean plus three standard deviations (SD) of plasma from 50 healthy blood donors. Titers above 11 AU/ml were considered positive.

### 2.4 Western blot analysis

Plasma or serum from patients and controls was investigated for the presence of FHR1 as described by Foltyn Zadura et al. (28) with the following adjustments: Samples were diluted 1:10, proteins were blocked with 5% dry milk in TBS+0.1% Tween and bound antibodies were detected with a rabbit anti-mouse IgG Fcy antibody conjugated with HRP (Jackson, Baltimore, MD, USA).

### 2.5 PCR of CFHR3/CFHR1 deletion

DNA was extracted from peripheral blood of 12 patients using the QIAamp DNA Blood Mini Kit from Qiagen (Hilden, Germany). The primers were designed using the SnapGene software (GSL Biotech LLC, San Diego, California, USA) and synthetized by Microsynth AG (Balgach, Switzerland). A deletion of CFHR1 and CFHR3 was confirmed by PCR amplification using specific primers. For CFHR1, the forward primer (fw) sequence was CCATCATCAATTTCAAAACCCGTGTCCTC and the reverse primer (rev) sequence was CAGATTTTAAGTGTCCTCACCACAAACACAC. This 1242-bp sized amplification product was detected by agarose gel electrophoresis. For CFHR3, the primer pair CFHR3 fw CAATTATTGGTAATGTGTGCACCCTGAAC and CFHR3 rev CTTGGTGCAAGATGACGAACCTCGGG resulted in a 1545-bp fragment on agarose gel electrophoresis. To verify the PCR process, we utilized two primer pairs targeting the FH region as positive controls, given the established presence of FH protein in all patients via ELISA and Western blot analyses. FH control 1 fw CTGTGGACATCCTGGAGATACTCCTTTTGG and FH control 1rev CAGAGAATAAGGGGGATAAAATAACACTGAGG produced a PCR product of 703 bp. The second primer pair, FH control 2 fw CATGGGTTATGAATACAGTGAAAGAGGAGATGC and FH control 2 rev GCAGCAGACCTCATCAAAAGCAAACC, was employed to amplify a fragment of 914 bp.

DNA amplification was performed according to the Q5 High-Fidelity PCR protocol (NEB, MA, USA). A 25-uL reaction volume contained 5 uL of 5x Q5 buffer (NEB, MA, USA), 1.25 uL of each primer at 10 mM, 0.5 µl of BioReady™ deoxyribonucleotide triphosphates (dNTP) mixture with each dNTP at 10 mM (Bioer, Japan), and 0.25 U of polymerase and 15µl distilled water. 35 cycles were used for DNA amplification, which was performed according to the Q5 PCR protocol provided by the manufacturer. 10 uL of amplified PCR product was analzed in a 1% (w/v) agarose gel under UV light using the Fusion FX Imaging system from Vilber (Witec AG, Switzerland).

### 2.6 Statistical analysis

Statistical analyses and graphical presentations were conducted using R software version 4.2.2 and GraphPad Prism (GraphPad Software, San Diego, CA, USA) versions 10.2.2 and 10.2.3. Univariate analyses were used to describe baseline characteristics. Data for continuous variables are presented as median with interquartile range (IQR). Categorical data are presented as frequency and percentage. Non-parametric-tests were used throughout, because of a lack of normal distribution in FH, FHR1 and anti-FH ELISA. Differences in protein levels and antibody titers were analyzed by a two‐sided Wilcoxon Rank-Sum Test.

Correlations of numerical variables were analyzed by Spearman’s correlation coefficient. Associations between categorical variables were analyzed using Fishers Exact Test.

Statistical significance was considered as *p ≤ 0.05, **p< 0.01, ***p< 0.001, ****p< 0.0001 respectively. Since we performed an explorative study without prespecified key hypothesis, type I error control was not implemented. Statistical tests are therefore used only for descriptive purposes. To evaluate the influence of outliers, we used Cook’s distance, calculated from a linear model as a diagnostic tool. Points with a Cook’s distance greater than $\frac{4}{n}$ (with n being the number of observations) were considered influential. For the calculation of the FHR1/FH ratio, we used the concentrations of these proteins measured in µg/ml. Although this method was chosen for simplicity, we also performed an analysis that incorporated the molar weights FH and FHR1, with respective molar masses of 155 kDa and 49 kDa. This adjustment recalibrated the FHR1 to FH ratios by a factor of approximately three, reflecting the threefold greater molar mass of FH compared to FHR1. Despite this recalibration to account for molar differences, both the distribution of the data and the outcomes of the statistical analyses remained consistent. For the calculation of FHR1/FH ratios, individuals without detectable FHR1 were excluded, to avoid dividing 0.

## 3 Results

### 3.1 Patient characteristics

FH and FHR1 levels were measured in plasma samples from 378 patients with SLE. Demographic and clinical characteristics have been descripted previously (27) and are summarized in **Table 1**.

**Table 1:** Demographic and clinical characteristics of patients with SLE and control groups.

	SLE n= 378*	NHP n=84	NHS n=84
Female, n (%)	324 (85.7)	30 (35.7)	42 (50)
Male, n (%)	54 (14.3)	54 (64.3)	42 (50)
Disease Classification at time of inclusion			
Number of ACR Criteria (1982), median (IQR)	5 (4–6)		
ACR Criteria (1982)			
Skin involvement †, n (%)	253/378 (66.9)		
Nasopharyngeal ulcers, n (%)	102/376 (27.1)		
Arthritis, n (%)	291/377 (77.2)		
Serositis, n (%)	119/376 (31.6)		
Renal disorder, n (%)	168/376 (44.7)		
Neurological disorder, n (%)	37/376 (9.8)		
Hematologic disorder, n (%)	240/377 (63.7)		
Immunological disorder, n (%)	316/374 (84.5)		
Ethnicity			
Caucasian, n (%)	280 (74.1)	83 (98.8)	80 (95.2)
African, n (%)	38 (10.1)	1 (1.2)	2 (2.4)
Asian, n (%)	37 (9.8)	0 (0.0)	1 (1.2)
IAA ‡, n (%)	18 (4.8)	0 (0.0)	0 (0.0)
Pacific Islander, n (%)	1 (0.3)	0 (0.0)	0 (0.0)
Other, n (%)	2 (0.5)	0 (0.0)	1 (1.2)
Unknown (%)	2 (0.4)	0 (0.0)	0 (0.0)
Age			
At blood sampling, median (IQR)	42 (32–54)	55 (36- 62)	45 (33.5–59)
Disease duration since Diagnosis of SLE (IQR)	5 (1–13)		
Disease Activity and Clinical Features			
Fever, n (%)	24/377 (6.4)		
Arthritis, n (%)	84/375 (22.4)		
Muco-cutaneous involvement ¶, n (%)	119/373 (31.9)		
Vasculitis, n (%)	8/377 (2.1)		
Serositis, n (%)	22/372 (5.9)		
CNS involvement §, n (%)	12/375 (3.2)		
Leukopenia, n (%)	53/372 (14.2)		
Thrombocytopenia, n (%)	31/373 (8.3)		
Anemia, n (%)	126/371(34.0)		
Elevated ESR #, n (%)	134/322 (41.6)		
Proteinuria, n (%)	56/298 (18.8)		
Hematuria, n (%)	63/340 (18.5)		
Low Complement, n (%)	112/341 (32.8)		
Anti-ds-DNA antibodies, n (%)	167/340 (49.1)		
Anti-Phospholipid antibodies, n (%)	59/183 (32.2)		

*n=378 unless otherwise stated; †skin involvement defined as malar rash, discoid rash or photosensitivity; ‡ IAA, Indigenous ancestry from the Americas; ¶ muco-cutaneous involvement defined as malar rash, mucosal ulcers or alopecia; §CNS involvement was defined as psychosis; seizure or organic brain syndrome; # ESR, erythrocyte sedimentation rate.

### 3.2 Plasma levels of FH and FHR1

**Figure 1:**
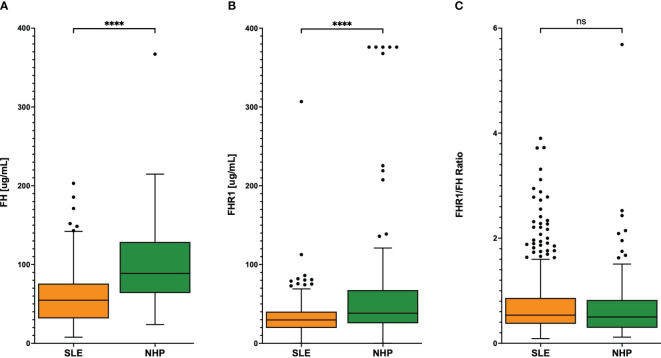
**(A)** FH and **(B)** FHR1 levels and **(C)** FHR1/FH ratio in SLE patients and controls. Graphs display Tukey’s boxplots with whisker lengths of 1.5x interquartile range. Outliers are shown as dots. Statistical significance was indicated as ****p< 0.0001 and ns, not significant.

Levels of FH and FHR1 were analyzed in plasma from 378 SLE patients as well as in 84 NHP and 84 NHS. FH levels were significantly higher in NHS compared to NHP (P< 0.0001; **Supplementary Figure 1**). Consequently, we focused on NHP for comparing protein levels between SLE patients and healthy controls. In healthy blood donors, levels of both FH and FHR1 were significantly higher compared to those in SLE plasma (P< 0.0001 respectively). This difference was also observed when excluding FHR1-defient patients from the analysis (P< 0.0001 respectively). Since FHRs are supposed to compete with FH for ligand binding (19) the ratio of these proteins may be an important indicator of potential regulatory imbalance as well. Therefore, we calculated the FHR1 to FH ratio to uncover any regulatory differences between SLE patients and healthy controls. However, no significant difference in FHR1/FH ratios was found between the two groups (P = 0.180; **Figure 1**). Regarding the distribution of the FH/FHR1 ratio in the SLE patients, we found a large number of outliers (n=38), assessed by the IQR method. Among these, 20 outliers were identified as influential by Cook’s distance. To investigate the statistical relevance of these outliers, we conducted a sensitivity analysis by excluding the 38 outliers, which confirmed that the findings remained robust, with still no significant difference between the SLE and NHP groups (Wilcoxon test p-value: 0.112).

### 3.3 No association of FH, FHR1 or FHR1/FH ratio with SLE disease activity

**Figure 2:**
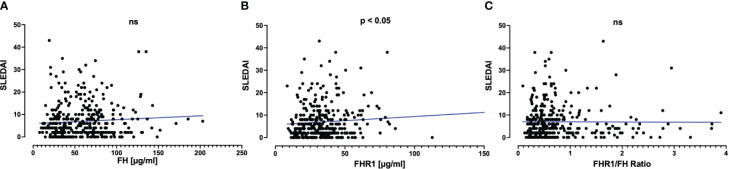
Correlation of **(A)** FH and **(B)** FHR1 levels and **(C)** FHR1/FH ratio with SLE disease activity. Graphs display scatterplots including a linear regression line in blue. Correlations with the SLEDAI Score were assessed using Spearman’s correlation coefficient, which was 0.09 (P=0.087) for FH, 0.12 (P=0.029) for FHR1, and 0.03 (P=0.63) for the FHR1/FH Ratio. Statistical significance was indicated as p ≤ 0.05 and ns, not significant.

Although the ratio between FHR1 and FH was similar when comparing SLE patients to healthy individuals, we hypothesized that variations in absolute levels of these proteins might correlate with disease activity in SLE as assessed by SLEDAI Score (**Figure 2**). Only a weak correlation was observed between FHR1 levels and disease activity (correlation coefficient = 0.12, P = 0.029), without significant correlations found for FH levels (P = 0.087) or the FHR1/FH ratio (P = 0.63). Further analyses were conducted to assess the correlation between FH, FHR1, and the FHR1/FH ratio with disease activity using the Wilcoxon test for active disease defined as PGA ≥ 1 and active disease defined as PGA ≥ 1 and SLEDAI > 6. These methods did not yield statistically significant correlations.

### 3.4 No association of FH, FHR1 or FHR1/FH ratio with clinical and laboratory parameters

We next examined whether variations in FH and FHR1 levels correlate with specific disease manifestations included in the SLEDAI Score. **Table 2** presents the p-values for correlations between individual protein levels and the FHR1/FH ratio, along with the presence of various disease manifestations. Beyond an association between FHR1 levels and anti-dsDNA (P=0.025), as well as anemia (P=0.043), no significant correlations were detected between FH, FHR1, or the FHR1/FH ratio and other clinical and laboratory manifestations of SLE.

**Table 2:** Correlation of clinical and laboratory features with FH, FHR1 and FHR1/FH ratio.

	FH level	FHR1 level	FHR1/FH Ratio
Clinical/Laboratory Feature	p-value	p-value	p-value
Fever	0.384 ns	0.073 ns	0.133 ns
Arthritis	0.205 ns	0.204 ns	0.218 ns
Skin involvement	0.661 ns	0.085 ns	0.026 ns
Vasculitis	0.678 ns	0.617 ns	0.964 ns
Serositis	0.614 ns	0.481 ns	0.191 ns
CNS involvement	0.813 ns	0.490 ns	0.168 ns
Leucopenia	0.524 ns	0.053 ns	0.096 ns
Thrombocytopenia	0.812 ns	0.217 ns	0.076 ns
Anemia	0.166 ns	0.184 ns	0.043 *
ESR†	0.715 ns	0.097 ns	0.719 ns
Proteinuria	0.447 ns	0.883 ns	0.210 ns
Hematuria	0.249 ns	0.080 ns	0.394 ns
Low Complement	0.226 ns	0.064 ns	0.799 ns
Anti-ds-DNA	0.097 ns	0.527 ns	0.025 *
APLA‡	0.161 ns	0.295 ns	0.992 ns

Clinical and laboratory features included in this analysis are derived from the SLEDAI score. †Erythrocyte sedimentation rate, ‡Antiphospholipid antibodies, P-values are derived from the Wilcoxon test. Statistical significance was considered as *p ≤ 0.05 and ns, Not significant (p > 0.05).

### 3.5 Deficiency of FHR1 is significantly increased in SLE patients

**Figure 3:**
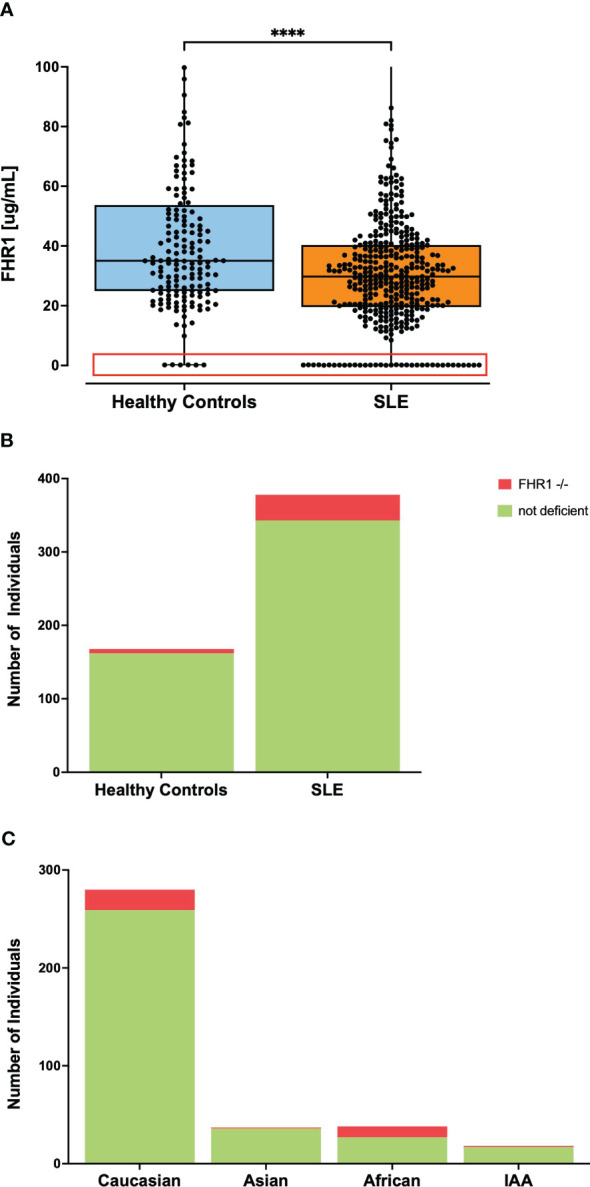
Frequency of individuals with undetectable FHR1 levels in ELISA. Part **(A)** shows levels of FHR1 with all data points represented as dots where the red box highlights individuals with undetectable FHR1 levels comprising 35 individuals in the SLE group and 6 in the healthy control group with the y-axis of the graph shortened for better visibility of the lower FHR1 values. Statistical significance was considered as ****p< 0.0001. Part **(B)** visualizes the percentage of individuals with undetectable FHR1 levels in ELISA, indicated in red, noting 9.26% of SLE patients and 3.57% in the control group (P= 0.0214). Part **(C)** visualizes the percentage of individuals with FHR1 deficiency across various ethnic subgroups in the SLE cohort. 28.9% of African patients, 2.7% of Asian patients, 5.6% of IAA, and 7.5% of Caucasian patients exhibited FHR1 deficiency. Due to small sample sizes, this graph does not contain information about the Pacific Islander subgroup.

It was noted that a substantial number of SLE patients exhibited FHR1 levels below the LOD in ELISA (**Figure 3A**). To compare the number of individuals with FHR1 levels below the LOD, plasma and serum from healthy blood donors were pooled, acknowledging that precise FHR1 values are not critical for this aspect of the study. A significant difference was observed with 35 (9.26%) of SLE patients having undetectable FHR1 levels compared to 6 (3.57%) in the control group (P = 0.021; **Figure 3B**). To ascertain the absence of FHR1 protein, levels ‘Below LOD’ were confirmed by Western blot (**Supplementary Figure 2**). Western blot analysis of serum samples using a mouse monoclonal antibody against FH (C18/3) revealed FH (150 kDa) and two differently glycosylated forms of CFHR1α and CFHR1β (37 and 42 kDa). Individuals with very low FHR1 levels, ranging from 10.5 to 20.2 µg/ml, exhibited detectable bands for FHR1. However, the lane representing an individual with undetectable FHR1 levels showed no bands, confirming the absence of FHR1 protein in such individuals. This demonstrates the sensitivity of Western blotting in detecting FHR1, with a complete absence of bands in individuals with FHR1 deficiency.

Usually, the absence of FHR1 indicates the common deletion of the genes encoding FHR1 and FHR3 (29). Therefore, as control, a genetic deficiency was confirmed by PCR in a subset of patients with undetectable FHR1 levels, as whole blood was not available for every patient. In this unselected subset, every SLE patient with FHR1 level below the LOD in ELISA was confirmed to have genetic deficiency, supporting previous observations that undetectable FHR1 levels in ELISA correspond to genetic deficiency (**Supplementary Figure 3**). Given the considerable variation in frequency of the common CFHR3–1 gene deletion across different ethnic groups (30), we analyzed the proportion of FHR1 deficiency in SLE patients by ethnicity, as shown in **Figure 3C**. In our SLE cohort, the proportion of FHR1-deficient individuals varied noticeably across different ethnic groups. Notably, 28.9% of patients with African ancestry exhibited FHR1-deficiency, contrasting markedly with just 2.7% of Asian and 5.6% of Indigenous ancestry from the Americas (IAA). The percentage in Caucasian patients was 7.5%. Our cohort included one Pacific Islander, who was FHR1-deficient. Since our control cohort consisted predominantly of Caucasian individuals (**Table 1**), we aimed to ensure that the observed significant difference in number of FHR1-deficient individuals was not driven by the ethnic diversity of our SLE cohort. For this, we obtained numbers of FHR1-deficiency in the general population across different ethnic groups from the literature (23, 30) to calculate the association between FHR1-deficiency and SLE susceptibility. Odds Ratios (ORs) are shown in **Table 3**. After stratification by ethnic groups, the consistent ORs still suggested an association between FHR1 deficiency and SLE, although the significance disappeared due to a reduced power, except for African SLE patients. For Caucasian patients, the odds ratio was 2.14 (95% CI: 0.892 - 6.04), for Asian patients it was 4.103 (95% CI: 0.128 - 51.937) and for African patients it was 4.587 (95% CI: 1.711 - 12.661). An OR for IAA could not be determined, as a 0% deficiency rate has been reported in the general population.

**Table 3:** Association between FHR1 deficiency and SLE.

Ethnicity		FHR1-/-	Not deficient	OR	95% CI
Caucasian	SLE*	21	259	2.14	0.892 - 6.04
	General Population†	6	157		
African	SLE*	11	28	4.587	1.711 - 12.661
	General Population (30)	9	103		
Asian	SLE*	1	36	4.103	0.128 - 51.937
	General Population (23)	2	280		
IAA	SLE*	1	17	NA	NA
	General Population (30)	0	29		

*The frequency of FHR1 deficiency in SLE patients was determined from those included in this study. †The frequency of FHR1 deficiency in the general Caucasian population was assessed using the blood donor group included in this study. Frequencies of FHR1 deficiency in the general population for African, Asian, and IAA groups were adopted from referenced literature.

NA, not available.

### 3.6 Influence of FHR1 deficiency on clinical presentation of SLE

**Figure 4:**
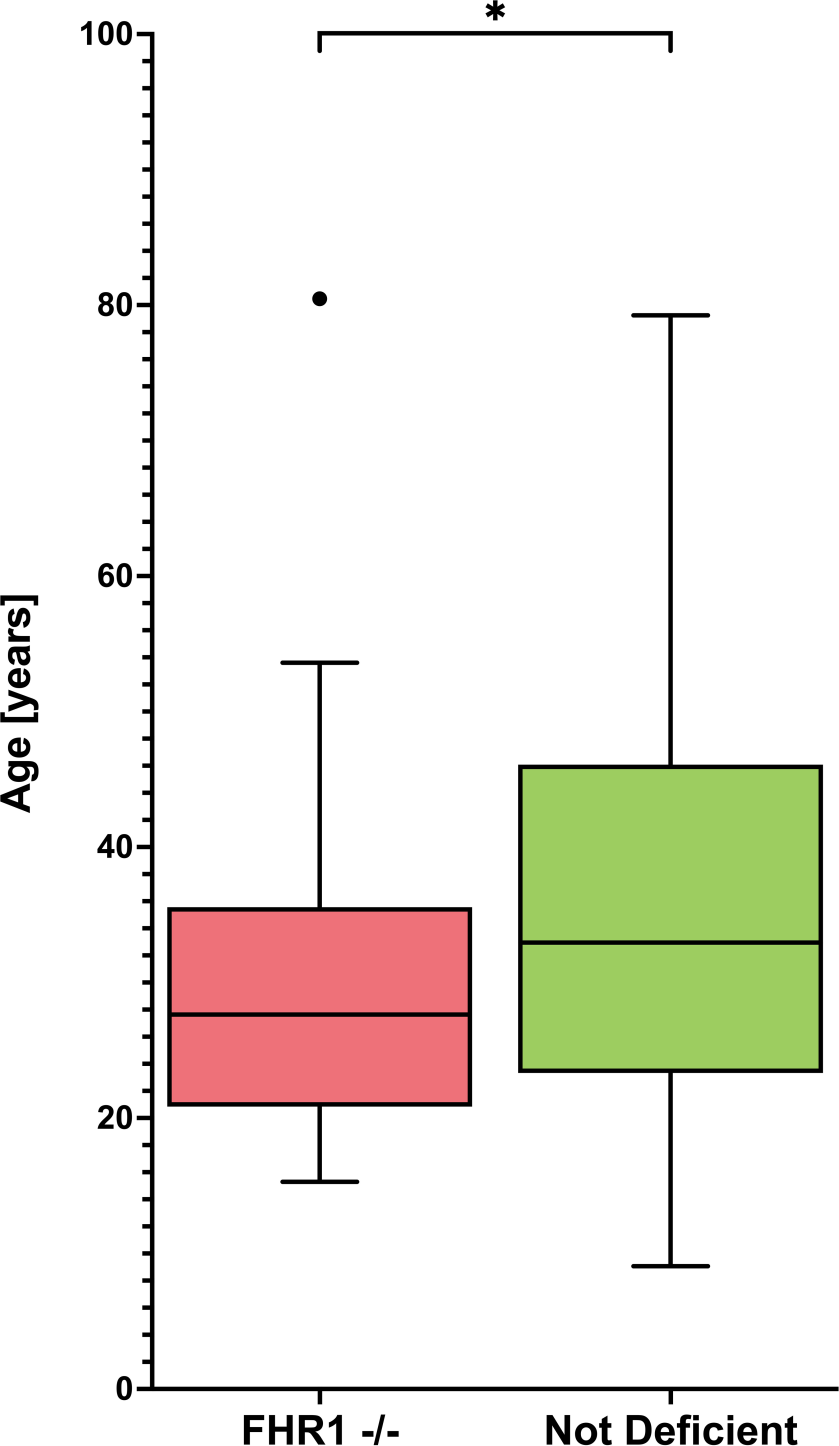
Age distribution of FHR1- deficient and not deficient SLE patients Graphs display Tukey’s boxplots with whisker lengths of 1.5x interquartile range. Outliers are shown as dots. The age distribution is shown for FHR1-deficient (FHR1 -/-) and not deficient SLE patients. Statistical significance between the groups was indicated as *p ≤ 0.05.

Given that our data indicated an association between FHR1-deficiency and SLE susceptibility, we next investigated whether the presence of FHR1-deficiency influences the clinical presentation of SLE. We therefore examined the relationship between deficiency and disease severity, measured by the number of ACR criteria met. However, our data showed no significant difference between patients with and without deficiency (P = 0.865). **Table 4** shows the association of FHR1-deficiency with clinical manifestations of SLE included in the 1982 ACR criteria. No specific disease manifestation was associated with the deficiency, except for hematologic disorder. However, our data analysis showed that patients with FHR1-deficiency were diagnosed with SLE at a significantly younger age compared to those without deficiency (P=0.048; **Figure 4**).

**Table 4:** Association of FHR1 deficiency with clinical manifestations of SLE.

ACR Criteria (1982)	P	OR	95% CI
Skin involvement †	0.577 ns	0.821	0.379 -1.844
Nasopharyngeal ulcers	0.691 ns	0.812	0.307 - 1.930
Arthritis	0.401 ns	0.715	0.315 - 1.744
Serositis	0.246 ns	1.578	0.708 - 3.430
Renal disorder	0.281ns	0.651	0.284 - 1.425
Neurological disorder	1 ns	0.877	0.163 - 3.05
Hematologic disorder	0.042 *	2.448	1.007 - 6.832
Immunological disorder	0.328 ns	1.991	0.589 – 10.532

†Skin involvement defined as malar rash, discoid rash or photosensitivity. P-values are derived from Fisher’s exact test. Statistical significance was considered as *p ≤ 0.05 and ns, Not significant (p > 0.05).

### 3.7 Relationship between FHR1 deficiency and anti-FH antibodies

The association between CFHR3–1 deficiency and anti-FH is well described in the context of atypical Hemolytic Uremic Syndrome (aHUS). In aHUS, the common deletion of CFHR3 and CFHR1 leads to the formation of autoantibodies directed against FH (31), which disrupt FH’s ability to protect host cells. This leads to complement attack against red blood cells, platelets, and endothelial cells, as seen in aHUS. Therefore, in aHUS, anti-FH autoantibodies are considered to be pathogenic, rather than the genetic deletion itself (32). Considering the significant link between CFHR3–1 deficiency and the presence of anti-FH in aHUS, we investigated whether a similar relationship exists within our SLE cohort.

**Figure 5:**
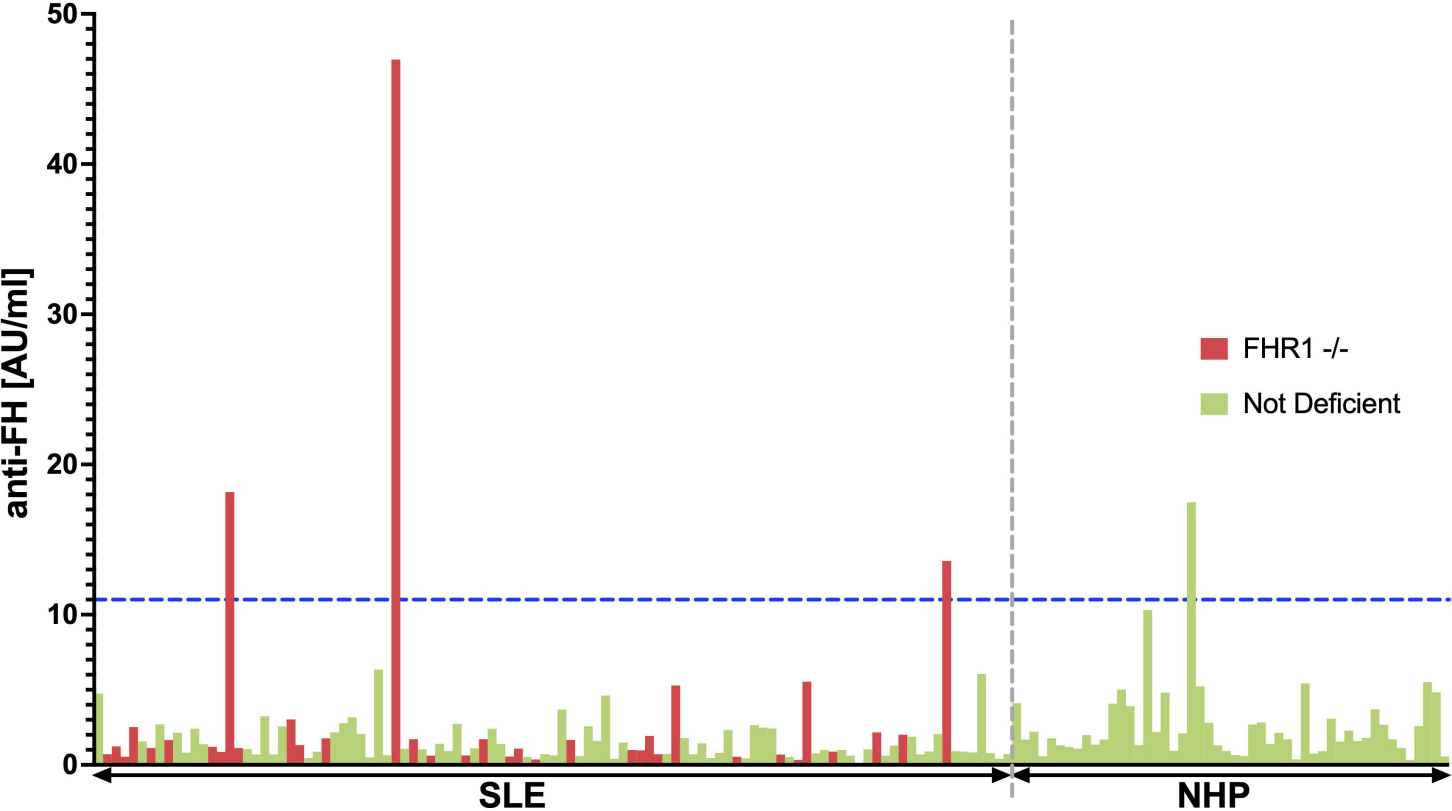
BarPlot of measured anti-FH levels. This bar plot visualizes anti-FH levels measured in AU/ml across the 35 FHR1-deficient SLE patients represented in red and 70 matched non-deficient SLE patients shown in green (1:2 matching). The cutoff value for anti-FH positivity, calculated using data from 50 healthy blood donors, is indicated by the blue dashed line. SLE patients and healthy individuals are separated by the gray dashed line.

The 35 FHR1-deficient SLE patients were matched with 70 non-deficient SLE patients (1:2 matching) based on sex, age, ethnic background, and disease activity (**Table 5**). **Figure 5** shows the observed anti-FH levels. Among the FHR1-deficient SLE patients, three out of 35 were identified as being anti-FH positive. One healthy control also tested positive for anti-FH. Levels of anti-FH did not differ significantly between deficient and non-deficient SLE patients (P = 0.666), but a significant association was found between anti-FH positivity and FHR1-deficiency (P = 0.039). All three SLE patients who tested positive for anti-FH had the genetic deficiency.

**Table 5:** Patient characteristics of SLE patients assessed for anti-FH measurement.

	SLE Plasma			Normal Human Plasma
	FHR1 -/- n=35	Not deficient n=70	P	Not deficient n=50
Sex = Male (%)	1 (2.9)	6 (8.6)	0.489	17 (34)
Age (median [IQR]	36 [30, 46]	42 [31, 49]	0.312	45 [30, 58.75]
Ethnicity				
Caucasian (%)	21 (60)	43 (61.4)		50 (100)
Asian (%)	1 (2.9)	3 (4.3)		
African (%)	11 (31.4)	22 (31.4)		
IAA (%)	1 (2.9)	2 (2.9)		
Pacific Islander (%)	1 (2.9)	0		
Other (%)	0	0		
SLEDAI Score (median [IQR]	4 [0, 10]	4 [1, 9.75]	0.847	

Characteristics of 35 FHR1-deficient SLE patients matched with non-deficient counterparts based on sex, age, ethnicity, and SLEDAI Score are presented. The table includes p-values derived from statistical tests used to compare age (Wilcoxon rank-sum test), sex (Fisher’s exact test), and disease activity (measured by SLEDAI Score using the Wilcoxon rank-sum test) between the two groups, demonstrating no significant differences.

However, in contrast to aHUS, where 63% of CFHR3–1 deficient patients are anti-FH positive (33), only 9% of FHR1 deficient SLE patients in our cohort tested positive for anti-FH. Patient characteristics of anti-FH positive SLE patients are shown in **Supplementary Table 1**. Only one of the three patients had hypocomplementemia and thrombocytopenia at the time of sampling, but none of the patients had clinical signs of TMA. Although the sample size is too small to draw firm conclusions, our results suggest that there is no relationship between anti-FH positivity in FHR1-deficient SLE patients and TMA.

## 4 Discussion

A critical gap remains in our understanding of the pathophysiological roles of the FHRs in various diseases (34). While a previous genetic study has highlighted the association between FHR1 deficiency and SLE susceptibility (23), the implications of variable protein levels of FH and FHR1, remained underexplored in a clinical setting (24). In this study, we aimed at characterizing FH and FHR1 protein levels in SLE patients and assessing their potential as biomarkers of the disease by comparing them to healthy controls and analyzing their relationship with clinical manifestations of SLE.

Although levels of both FH and FHR1 were significantly higher in healthy blood donors than in SLE patients, the FHR1/FH ratio remained similar between the two groups (**Figure 1**), suggesting that there is no regulatory imbalance of these proteins in SLE patients. The significantly higher levels of both proteins in healthy controls may be attributed to more intensive continuous consumption due to ongoing complement activation in SLE patients leading to a shift in the balance between production and recycling, but we cannot exclude effects due to differences in storage times of the plasma samples at -80°C. We observed no association between the FHR1/FH ratios and SLE disease activity and only a weak correlation between FHR1 levels and disease activity (**Figure 2**). Additionally, the lack of a clear correlation of FHR1 levels and the FHR1/FH ratios with clinical and laboratory features (**Table 2**) indicates that FHR1 levels may not affect the disease expression.

However, the significant difference in the prevalence of FHR1 deficiency between SLE patients and controls (**Figure 3B**), with a consistent trend across all ethnic subgroups (**Figure 3C**, **Table 3**), supports the notion that the lack of FHR1 is a risk factor for SLE. This hypothesis is further supported by the observation that patients with FHR1 deficiency are diagnosed with SLE at a significantly younger age compared to non-deficient individuals.

To our knowledge, there is only one work to date that has studied the association of FHR1 deficiency with SLE (23). Compared to this study, the frequency of FHR1 deficiency among SLE patients observed in our study is almost two times higher across different ethnic groups (Caucasian: 7.5% vs. 5.2%, African: 28.9% vs. 15.7%, and Asian: 2.7% vs. 1.1%), while we also we could not establish an association between the presence of FHR1 deficiency and disease severity or distinct disease manifestations (**Table 4**).

The observed lack of FHR1 most likely is due to a common genetic deficiency of the genes encoding FHR3 and FHR1 (29), although this common deletion formally was not demonstrated in all patients investigated here. In this context, we cannot exclude the possibility that the lack of FHR3 is crucial for our observations. However, we believe that the absence of FHR1 is the primary driver for the associations seen in our study. This assumption is based on the fact that FHR1 more closely resembles FH with regard to the C-terminal domains of FH that mediate the binding of FH to host surfaces, even though the sites of regulatory activities might differ between FH and FHR1 (26). Additionally, available data on the regulatory function of FHR1 appear to be more solid than for FHR3, and quantitatively, FHR1 outweighs FHR3 levels by a factor of at least 100 (34). However, more data is required to exclude a role of FHR3 in SLE.

In aHUS the common deletion of CFHR3 and CFHR1 seems to drive the formation of autoantibodies directed against FH, most likely because the C-terminal SCRs of FHR1 is involved in the induction of tolerance to a cryptic epitope in FH’s C-terminal region (31). These anti-FHs then disrupt FH’s ability to protect host cells, leading to the clinical manifestations of the disease such as microangiopathic hemolytic anemia, acute renal failure, and thrombocytopenia (32).

Intriguingly, all three SLE patients who tested positive for anti-FH had the genetic deficiency. While the clinical impact may be small, the association between FHR1 deficiency and anti-FH positivity provides valuable insights into the underlying mechanisms of autoantibody formation in SLE. The finding supports the hypothesis that FHR1 is involved in the maintenance of immunological tolerance to specific regions of FH.

However, in contrast to aHUS, where 63% of CFHR3–1 deficient patients are anti-FH positive (33), only 9% of FHR1-deficient SLE patients in our cohort tested positive for anti-FH, indicating a limited pathogenic role of these autoantibodies in FHR1-deficient SLE patients. This hypothesis is supported by the observation, that only one of the three FHR1-deficient, anti-FH positive SLE patients had hypocomplementemia, and none of them had clinical signs of TMA (**Supplementary Table 1**). It is noteworthy that an identical mechanism of anti-FH in aHUS and SLE patients is not necessarily expected, since it has been shown that anti-FHs in SLE interact with different regions of FH (28).

In aHUS, mainly autoantibodies to FH of the IgG class have been described (35–37), while anti-FH autoantibodies of other immunoglobulin classes are less frequently reported. Cugno et al. detected IgM anti-factor H autoantibodies in seven of 186 (3.8%) patients with aHUS, without association between anti-factor H IgM and homozygous deletions of CFHR3-CFHR1 (38). To the best of our knowledge, anti-FH autoantibodies of classes other than IgG have not been reported yet in SLE, but such an analysis would be an interesting avenue for future studies.

The reasons why the absence of FHR1 increases the risk for the development of SLE remain obscure. FHR1 is considered to regulate the regulator (FH) (10). Thus, the absence of FHR1 should increase the potency of FH and lead to reduced complement activation due to enhanced control. Considering the CS as an important mediator of inflammation in SLE, our observation is contrary to our expectation. There are two possible reasons for this. First, the role of FHR1 in SLE may differ from the current understanding as a regulator of FH, although there are solid data underscoring the complement activation properties of the FHRs (18, 19). Second, contrary to the prevailing view, complement activation might be beneficial rather than harmful in SLE. This theory aligns with observations that deficiencies of early proteins of the CP also increase the risk of SLE (39). It may be that the absence of FHR1 leads to increased control of protective complement activation or modulate specific FH functions that remain undefined. However, apart from regulating the common C3b-amplification loop where all pathways of complement activation converge, a role for FH within the (early) classical pathway is not well established yet (40).

In conclusion, our study highlights the complexity of the roles of FHR1 in SLE. While the lack of FHR1 seems to be a risk factor for SLE, its levels do not appear to correlate with disease severity or specific clinical manifestations. The potential pathogenic role of FHR1 deficiency in SLE requires further investigation, particularly in relation to its interaction with FH and other complement regulatory proteins. Understanding the precise mechanisms by which FHR1 influences SLE development and progression could open new avenues for diagnostic and therapeutic strategies. More comprehensive studies are necessary to elucidate these mechanisms and to determine the full impact of FHR1 and related proteins.

## Data availability statement

The raw data supporting the conclusions of this article will be made available by the authors, without undue reservation.

## Ethics statement

The studies involving human participants were reviewed and approved by Swissethics (ethical committee of the Canton Vaud, Switzerland Ref. No. 2017-01434). The studies were conducted in accordance with the local legislation and institutional requirements. The participants provided their written informed consent to participate in this study.

## Author contributions

JSK: Writing – original draft, Data curation, Formal Analysis, Software, Validation, Visualization. JK: Writing – original draft, Methodology. DD: Writing – original draft, Methodology. LI: Writing – original draft, Data curation. CC: Writing – original draft, Data curation, Supervision. UH: Writing – original draft, Data curation. CR: Writing – original draft, Data curation, Project administration. MT: Writing – review & editing, Conceptualization, Funding acquisition, Methodology, Project administration, Resources, Supervision.
