# Supplementary material for: Factor H-related protein 1 in systemic lupus erythematosus
Source: Front Immunol. 2024 Jul 29;15:1447991. doi: 10.3389/fimmu.2024.1447991 (PMC11317429; doi:10.3389/fimmu.2024.1447991)
Supplement: Supplementary file 1 [file DataSheet_1.docx]

Supplementary Material

Supplementary Figure 1 | (A) FH and (B) FHR1 Levels and (C) FHR1/FH Ratio in NHS and NHP. Graphs display Tukey’s boxplots with whisker lengths of 1.5x interquartile range. Outliers are depicted as dots. Statistical significance was indicated as ****p < 0.0001 and ns, not significant.

Supplementary Figure 2 | Western blot analysis of serum samples from individuals with very low FHR1 levels using a mouse monoclonal antibody against FH (C18/3) that recognizes FH (150 kDA) and the two differently glycosylated forms of CFHR1⍺ and CFHR1β (37 and 42 kDa). Lane FHR1-/- shows a serum sample of an individual with undetectable FHR1 levels in serum. Levels of FHR1 in lane 1–7 were as follows: Lane 1: 10.5 µg/ml, Lane 2: 11.2 µg/ml, Lane 3 12.9 µg/ml, Lane 4: 16.6 µg/ml, Lane 5: 17.6 µg/ml, Lane 6: 19.8 µg/ml, and Lane 7: 20.2 µg/ml. FH shows a double band, due to a cleavage fragment of FH generated by plasma proteases, a known phenomenon that is enhanced after freeze- thawing (1). This Western blot analysis demonstrates that FHR1 is detected very sensitively by Western blotting. Only in the individual with CFHR1 deficiency the bands are completely absent.

Supplementary Figure 3 | Confirmation of genetic deficiency in a subset of patients by PCR. The figure shows the amplification products for CFHR1 and CFHR3. Individuals highlighted in red indicate levels that are below the LOD of the ELISA. Since the common CFHR3 – CFHR1 deletion results in the absence of both genes, the patients with undetectable FHR1 levels lack the amplification products for both CFHR3 and CFHR1.

Supplementary Table 1| Patient Characteristics of Anti-FH positive SLE Patients

|  | Patient 1 | Patient 2 | Patient 3 |
| --- | --- | --- | --- |
| Sex | female | female | female |
| Age [years] | 46 | 26 | 29 |
| Renal Involvement (ACR Criteria) | no | no | NA |
| Number of ACR Criteria | 5 | 4 | 6 |
| Systolic blood pressure [mmHg] | NA | NA | 120 |
| Diastolic blood pressure [mmHg] | NA | NA | 74 |
| Active smoking | no | yes | yes |
| SLEDAI Score | 4 | 4 | 15 |
| Thrombocytopenia | no | no | yes |
| Hematuria | NA | no | no |
| Hemoglobin [g/L] | 136 | 128 | 121 |
| Platelets [x10^9^/L] | 232 | 165 | 79 |
| Creatinine [µmol/L] | 74 | 45 | 76 |
| C3 [g/L] | NA | 0.82 | 0.18 |

This table presents the characteristics of CFHR1-deficient SLE patients who tested positive for anti-FH autoantibodies, as detected by ELISA. Given the known association of anti-FH antibodies with CFHR3-1 deficiency primarily in aHUS patients, these characteristics were analyzed with particular attention to signs of TMA.

1. Fontaine M, Demares MJ, Koistinen V, Day AJ, Davrinche C, Sim RB, et al. Truncated forms of human complement factor H. Biochem J. 1989;258(3):927-30.
